# Review of St. Paul's Hospital Millennium Medical College Radiology Program: Stakeholders' Viewpoints

**DOI:** 10.4314/ejhs.v34i1.2S

**Published:** 2024-10

**Authors:** Kumlachew Abate Mekonen, Alemayehu Bedane, Ashenafi Aberra Buser, Tesfaye Kebede, Shimels Hussien Mohammed

**Affiliations:** 1 Department of Radiology and Medical Radiologic Technology, School of Medicine, St. Paul's Hospital Millennium Medical College, Addis Ababa, Ethiopia; 2 Department of Radiology, School of Medicine, Addis Ababa University, Addis Ababa, Ethiopia; 3 Department of Public Health, School of Public Health, St. Paul's Hospital Millennium Medical College, Addis Ababa, Ethiopia

## Abstract

**Background:**

St. Paul's Hospital Millennium Medical College (SPHMMC) has offered radiology specialty training for a decade. To mark its 10th anniversary and assess the program's status, this study aimed to gather stakeholders' perspectives on its functioning.

**Methods:**

The assessment was conducted from June 15 to July 5, 2024, using a stratified cross-sectional study design. A total of 211 participants were recruited through exhaustive sampling from alumni, faculty, students, referring physicians, and patients. Data were collected via web-based and paper-based questionnaires, where stakeholders rated the core functions of the SPHMMC radiology program (curriculum, teaching, research, and imaging practices) on a 5-point scale and provided additional feedback.

**Results:**

Overall, 78% of stakeholders regarded the program's curriculum as highly relevant. However, teaching quality and research were viewed negatively, with only 19% and 11% of radiologists rating these aspects positively. Referring physicians considered radiology reports relevant (68%) but often found them incomplete and untimely (73%), with only 2% deemed timely. Over 80% of patients expressed dissatisfaction with the imaging services and were unwilling to recommend them.

**Conclusions:**

While positive perceptions of the curriculum and imaging services suggest program strengths, the negative feedback on teaching and research quality indicates the need for improvements to maintain SPHMMC's commitment to quality education and services.

## Introduction

St. Paul's Hospital Millennium Medical College (SPHMMC) is a leading provider of clinical services and medical training in Ethiopia (1,2,3,4). Patients from across the country rely on SPHMMC for imaging services. In 2014, the College established a radiology residency program aimed at training specialists at both the specialty and sub-specialty levels. To date, the program has produced over 110 radiologists who are now practicing in various capacities across different regions.

The radiology training program equips trainees with essential skills in imaging technologies, including computed tomography (CT), ultrasound (US), magnetic resonance imaging (MRI), and X-rays. These modalities are crucial for patient management and routine medical check-ups, positioning radiology as a fundamental aspect of modern healthcare (5-7). The program also emphasizes research and analytical skills, enabling graduates to contribute to the advancement of medical knowledge, particularly in radiology.

Periodic evaluations of educational programs are vital for ensuring their effectiveness and alignment with educational and service goals (8-10). Various evaluation approaches exist, such as formal effectiveness evaluations, program reviews, and performance audits (11). Program reviews are particularly useful when the objective is to quickly assess educational programs within limited time and resources (8,10,11). Such reviews often capture stakeholders' opinions on program strengths, weaknesses, opportunities, and threats, including feedback from students, teachers, patients, and management on curriculum relevance and teaching methods (1,2,11).

Despite operating since 2014, a systematic review of SPHMMC's radiology residency program has not been conducted. Stakeholders' perspectives on the program's strengths and weaknesses—particularly regarding curriculum relevance and patient satisfaction—remain largely unexamined.

As SPHMMC's Radiology Residency Program celebrates its 10th anniversary in October 2024, this study aims to bridge the evidence gap by gathering insights from current residents, fellows, patients, physicians, and academic staff regarding the curriculum's appropriateness, teaching quality, research emphasis, and imaging services efficiency. The findings will inform future evaluations and amendments to ensure the continued delivery of high-quality education, research, and clinical services.

## Materials and Methods

**Study setting and participants**: The study took place at SPHMMC, Addis Ababa, Ethiopia, from June 15 to July 5, 2024. Participants included stakeholders from the radiology program, focusing primarily on internal stakeholders, such as current faculty, external examiners, residents, department heads, and alumni. Feedback was also sought from patients, provosts, and referring physicians.

**Study design, sample size, and sampling approach**: This cross-sectional study calculated the sample size using a one-proportion formula (12). Based on previous satisfaction levels and a target population of 300, a minimum sample size of 140 was determined. Given the small target population, exhaustive sampling was employed to invite all eligible participants via web-based forms. To enhance response rates, key contacts among stakeholders were engaged for follow-ups.

### Variables and measurement

Sociodemographic variables: Key sociodemographic data included sex, age, residence, education level, and job status.

Stakeholder views: The study focused on assessing the following variables:

Curriculum relevance: Stakeholders' perceptions of whether the curriculum adequately equips graduates with necessary skills.

Teaching methodology quality: Evaluations of the effectiveness of teaching methods.

Research emphasis and preparedness: Assessments of the program's focus on research and its influence on graduates' readiness for research engagement.

Clinical service provision: Evaluations of the quality of imaging services and their alignment with physicians' needs.

Patient perspectives: Feedback on imaging services, including timeliness and quality compared to other facilities.

**Data collection**: Data were collected from June 15 to July 5, 2024, using both paper and electronic tools to accommodate varying levels of internet access. Stakeholder views were measured on a 5-point Likert scale, supplemented with open-ended questions for additional feedback.

**Statistical management and analysis**: Data from electronic questionnaires were processed in Excel and analyzed using SPSS-26, applying descriptive statistics to summarize findings. Responses were categorized to assess favorable and unfavorable views.

**Ethical considerations**: The study received ethical approval from the SPHMMC Institutional Review Board. Participants were informed of their rights and the voluntary nature of participation, with no personal identifiers collected.

## Results

**Sociodemographic characteristics of participants**: A total of 211 individuals responded to the invitation, with 72.5% identifying as male and 27.5% as female. Participants' ages ranged from 10 to 74 years, with 50% aged 30-40. Approximately 73% were from Addis Ababa. The stakeholder distribution included alumni (37.0%), current residents and fellows (6.6%), referring clinicians (29.9%), academic staff (7.1%), and patients (19.5%). Over 90% held university degrees, predominantly in medicine (Table 1).

**Table 1:** Sociodemographic characteristics of study participants (N=211)

Variable and Category	Frequency (%)
**Sex**	
Male	153 (72.5)
Female	58 (27.5)
Age (years)	
≤30	43 (20.6)
31-34	64 (30.4)
35-39	41 (19.6)
≥40	62 (29.4)
**Residence**	
Addis Ababa	153 (72.6)
Outside Addis Ababa	58 (27.4)
**Stakeholder Category**	
Alumni	78 (37.0)
Radiology resident	14 (6.6)
Referring physician	63 (29.9)
Radiology faculty	15 (7.1)
Patient	41 (19.5)
**Current Job**	
Associate professor	9 (4.3)
Assistant professor	70 (33.2)
Radiology Specialist	50 (23.6)
Resident and fellow	36 (17.1)
Lecturer	5 (2.4)
Teacher	6 (2.8)
Management	6 (2.8)
Trader	17 (8.1)
Unemployed	12 (5.7)
**Education Level**	
MD (Specialty)	120 (59.1)
MD (Sub-specialty)	22 (10.8)
MD	20 (9.9)
MSc/PhD	18 (8.8)
BSc	3 (1.5)
High School	6 (3.0)
Primary	14 (6.9)

**Figure 4:**
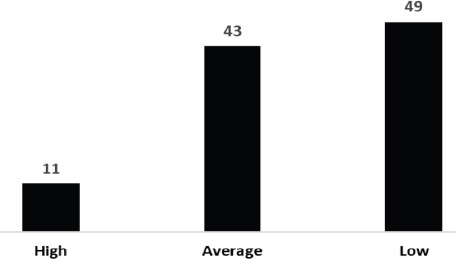
Stakeholder's Views on Research Quality (%)

**Views on research quality**: Figure 4 presents stakeholder opinions on the emphasis and quality of research conducted in the SPHMMC radiology department. The findings indicate a predominantly negative perception, with only 11% of respondents rating the quality of research as high. In contrast, 49% viewed it as low quality, while 43% rated it as average.

**Figure 5 A & B:**
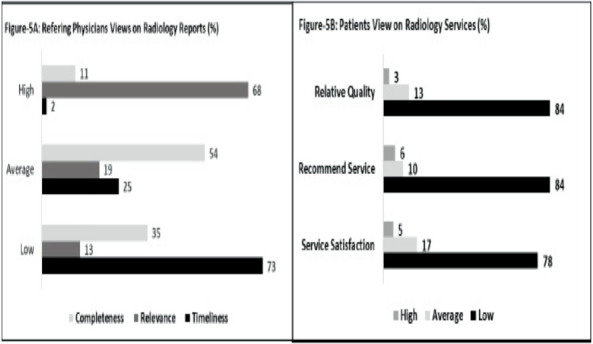
Physicians view on radiology report and patients view on the services

**Views on imaging services quality**: The perspectives of physicians who ordered CT, MRI, US, and X-ray imaging are shown in Figure 5A. A majority (68%) rated the relevance of imaging reports in patient care as high. However, opinions regarding the timeliness of these reports were overwhelmingly negative; 73% felt the reports were often untimely, with only 2% viewing them as timely. Additionally, over half of the participants (54%) considered the completeness of radiology reports to be average, while 35% deemed them usually incomplete and 11% as usually complete. Figure 5B captures patient feedback on imaging services, reflecting a generally negative sentiment across three key areas: overall service quality, relative quality compared to other centers, and willingness to recommend the service to others. A notable 78% of patients expressed dissatisfaction with the overall quality of imaging services, with only 5% satisfied. Additionally, 84% rated both the relative quality and likelihood of recommending the service as low.

## Discussion

This study gathered insights from various stakeholders, including faculty, residents, alumni, referring clinicians, and patients. The findings reveal significant strengths and areas for improvement within the program. Most stakeholders viewed the curriculum favorably, suggesting it effectively equips graduates with the necessary radiology skills and meets student needs. This positive perception should be leveraged to adapt to evolving clinical practices and technological advancements (5-7). However, a stark contrast exists between the curriculum's strengths and the perceived deficiencies in teaching methods and research quality, likely due to resource constraints and faculty capacity issues.

While many radiologists appreciated the practical, case-based training provided across various imaging modalities, there were widespread concerns about the overall quality of teaching methods. Stakeholders cited factors contributing to this low quality, including an insufficient number of instructors relative to the number of residents, limited availability of senior radiologists for consultation, and a lack of structured, consistent curriculum. Addressing these issues could lead to improved educational outcomes and enhanced perceptions of teaching quality. Research supports that continuous teacher development, and the integration of modern pedagogical methods are critical for enhancing teaching quality (13,15-17).

The majority of stakeholders also rated the quality of research at SPHMMC's radiology department poorly, a sentiment echoed by alumni, faculty, and current radiologists. Factors influencing this perception included an excessive focus on clinical services, minimal emphasis on research beyond the final-year thesis, and a lack of organized support for research activities. While improving these conditions could enhance the department's research focus, it's essential to recognize that these issues are not unique to radiology and are common in resource-limited settings. Previous studies indicate that inadequate research practices are prevalent in many developing countries, where clinical practice often overshadows the importance of fostering a research-oriented environment (3,18). The perspectives of referring physicians and patients on imaging services provide critical insights into the clinical service quality of the SPHMMC radiology department. While referring physicians recognized the relevance of imaging services for diagnosis and disease management, they expressed significant concerns regarding the timeliness, completeness, and overall quality of imaging reports. Patients also reported high levels of dissatisfaction with the timeliness and quality of imaging services, with many unwilling to recommend SPHMMC to others. Such feedback suggests the department is struggling to meet its clinical mission effectively.

Factors contributing to the dissatisfaction reported by radiologists, patients, and referring physicians included high patient volume, limited access to senior radiologists, outdated equipment, frequent downtimes of the internet and imaging systems, and insufficient resources for image archiving. Addressing these challenges, along with fostering ongoing communication among patients, physicians, and radiologists, could help reduce waiting times and improve stakeholder satisfaction with imaging services (13,15).

One of the study's strengths was its attempt to include a diverse range of stakeholders, providing a comprehensive view of the program's current state. Additionally, the use of web-based questionnaires likely reduced social desirability bias. However, the study has limitations, including potential bias in self-reported data and the absence of insights from key program figures such as the head of the radiology department and school deans. Future research should aim to include a broader range of stakeholders, employ mixed methods approaches, and incorporate performance measures to better assess service quality and resident outcomes over time.

In conclusion, this study offers a detailed review of the SPHMMC radiology program, highlighting both its strengths and areas requiring improvement. The positive feedback on the curriculum's relevance and the importance of imaging modalities in clinical service provision underscores the program's vital role in radiology education and patient care in Ethiopia. However, the negative views on teaching methodologies, research practices, and clinical imaging services highlight the urgent need for systematic improvement initiatives. Recommendations for enhancing the program include refining teaching methodologies, strengthening research capabilities, establishing a patient feedback system, optimizing workflows for improved service timeliness, exploring teleradiology and artificial intelligence, and developing both local and international partnerships. By addressing identified gaps while leveraging its strengths, SPHMMC can enhance its radiology program, ensuring it remains a leading institution in medical radiology education, research, and service provision.

## Figures and Tables

**Figure-1:**
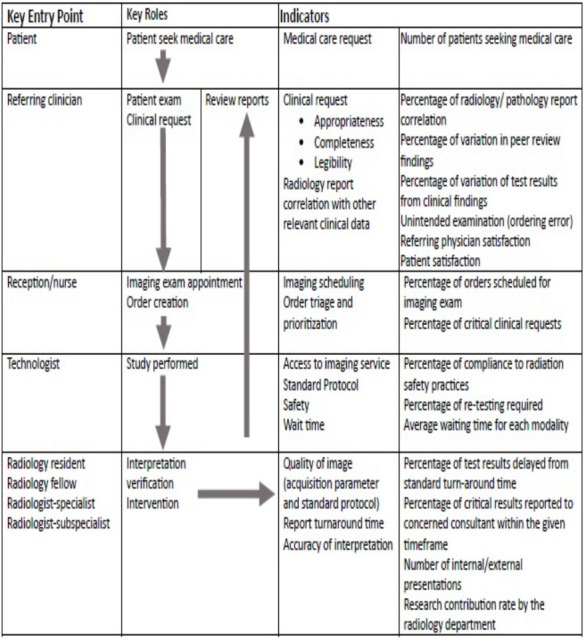
Flow Map of Radiology Clinical Services, SPHMMC

**Figure 3:**
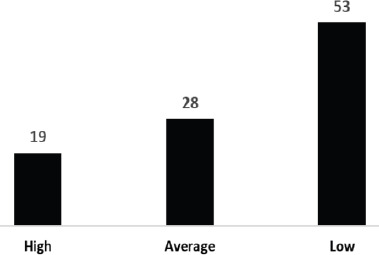
Stakeholder's Views on Teaching Methods Quality (%)
